# Access to COVID-19 Vaccination during the Pandemic in the Informal Settlements of Rome

**DOI:** 10.3390/ijerph19020719

**Published:** 2022-01-10

**Authors:** Enrico Bentivegna, Silvia Di Meo, Anita Carriero, Nadia Capriotti, Alberto Barbieri, Paolo Martelletti

**Affiliations:** 1 Internal Medicine and Emergency Medicine, Sant’ Andrea Hospital, Sapienza University, 00189 Rome, Italy; 2 Medici per i Diritti Umani–MEDU (Doctors for Human Rights–Italy) NPO, 00185 Rome, Italy; meducamper@mediciperidirittiumani.org (A.C.); 6nadiacapriotti@gmail.com (N.C.); alberto.barbieri@mediciperidirittiumani.org (A.B.); 3 DISFOR–Department of Education Sciences, University of Genoa, 16128 Genoa, Italy; silvia.dimeo4@gmail.com; 4 Department of Clinical and Molecular Medicine, Sapienza University of Rome, 00189 Rome, Italy; paolo.martelletti@uniroma1.it

**Keywords:** COVID-19 vaccine, vaccination campaign, public health, migrants, homeless

## Abstract

With the advent of vaccines, the world has a chance to see a real end to the COVID-19 pandemic. To make this possible, however, it is necessary that all groups of people are considered. Contexts of informal settlements and populations such as the homeless and migrants are often forgotten by vaccination campaigns. In this study, carried out as a result of a collaboration with MEDU, a non-profit association aimed at bringing healthcare to vulnerable populations, we provide important data related to the vaccination campaign carried out in the informal settlements of Rome. The objectives of this work are to (1) evaluate vaccination coverage in these contexts, (2) assess the gap with the vaccination coverage of the Italian population and try to hypothesize the causes, and (3) provide recommendations for how humanitarian associations can respond to reduce this gap. We observed important differences in vaccination coverage depending on the type of settlement. The percentage of vaccinated people in these contexts at the beginning of October range between 14.4% and 55.5%, underlining an important gap with the vaccination rate of Italy’s population, which is close to 80%. The data also show that particular attention must be paid to the transiting and irregular people as they are more at risk for a lack of access to vaccination. With this study, in which we provide recommendations that integrate MEDU’s fieldwork experience with the advice of the *Framework* report, we hope we can help those who work in similar contexts, to carry out a fair and effective vaccination campaign.

## 1. Introduction

### 1.1. Vulnerable People during the Pandemic

With the advent of vaccines, the world has a chance to see a real end to this pandemic. To make this possible, however, it is necessary that all categories of people are considered, and that representation is given to people based on their vulnerability and risk of contagion [1]. It is a shared opinion within the scientific community that the eradication of the pandemic must necessarily involve vaccination of the entire world population [2]. Despite these shared principles, residents in informal settlements, the homeless, and migrants, are often forgotten by vaccination campaigns. As the pandemic progresses, countries are increasingly facing an emergency with preventive measures to protect their citizens. Nevertheless, there is a lack of corresponding commitment to protect vulnerable populations such as migrants, the homeless, and residents in informal settlements [3]. These categories of people often fear and face legal threats, have limited access to public healthcare [4,5], and have a hard time fulfilling social distancing measures [6]. Countries that obtain COVID-19 Global Access (COVAX) vaccines, in collaboration with International Organization for Migration and Médecins sans Frontières, should plan to include refugees, asylum seekers, homeless populations, and foreign migrants as high-risk populations thereby in need of greater protection during the pandemic [7,8]. There are many calls for international organizations and advocacy groups working with asylum seekers and migrants to prevent these groups from being left behind in the fight against the pandemic. Despite this fact, it is not clear how this collaboration can occur, and there is still no effective coordination. It has been proposed that asylum seekers and refugees be placed in “at-risk populations” in order to prioritize them in vaccination, but the responses from the government are still not satisfactory [8]. Historically, global health response in emergency conditions exacerbates social inequalities, putting these populations at higher risk for physical and mental health crises [7,9].

### 1.2. Ethical Distribution of Vaccines

Emanuel et al. [10] suggest the following principles as a guide for an ethical distribution of vaccines within states: (1) limit damage and benefit people, (2) give priority to the most disadvantaged categories, and (3) ensure equal moral concern for all. According to these principles, residents in informal settlements and people at an economic disadvantage or not able to access public healthcare should be prioritized for vaccines because of their extreme vulnerability in the event of infection by the virus.

Agencies such as the International Organization for Migration (IOM), World Health Organization (WHO), and United Nations have repeatedly stressed the need to give particular consideration to vulnerable communities during the pandemic and vaccination campaign [11,12]. Stateless persons and people with unstable residence status or without ID documents usually only have access to emergency care. In an exceptional situation such as the pandemic, their vulnerability increases.

It should also be considered that the real number of stateless people is often unknown; therefore, it is unlikely that states with a large number of people without identification will be able to complete the vaccination campaign by this year [13,14].

Some steps have already been taken: A complaint from the UN Network on Migration urged states to provide vaccines to all migrant populations regardless of their condition [15]. In addition, Gavi, the global vaccine alliance, has signed a document with the IOM [16] to help migrants and displaced people during the pandemic. Nonetheless, detailed procedures are still missing, and improvements remain difficult.

The National Academies Press recently produced a report called *Framework* *for Equitable Allocation of COVID-19 Vaccine* (*Framework*) in which it describes specific recommendations for a fair vaccination campaign [17]. Health agencies should prioritize the *Framework* recommendations since it is currently the best available documentation, containing useful elements for the vaccination campaign among the most vulnerable populations such as refugees, migrants, the homeless, and stateless persons. It specifies how health agencies should know and take into account cultural differences and beliefs about community vaccination practices in order to reduce hostilities and foster an effective vaccination campaign.

### 1.3. Population of Informal Settlements

Informal settlements often host all the previously mentioned high-risk categories. Residents in these facilities are a heterogeneous group of people with great variability in attitudes toward vaccination. In most cases, they are immigrants, with a very diverse range of social status, education, and knowledge of the local language. This heterogeneity, combined with the general indifference of the national health system to these populations, further complicates the planning of a clear education and vaccination project [18]. Migrants are often young workers and therefore outside the risk groups according to the World Health Organization’s “values framework” [19]. Nevertheless, they often live in crowded accommodations without access to personal protective equipment (PPE) or healthcare. They also generally have low-income jobs where it is impossible to maintain social distancing and are subject to a high prevalence of diseases such as diabetes or obesity that favor COVID-19 complications [20,21].

### 1.4. Distrust and Collaboration

Social barriers are one of the major obstacles to vaccination in contexts such as informal settlements. Many migrants lack confidence in institutions and fear punitive action if they are identified. In a recent editorial, the British Medical Journal (BMJ) [14] stressed the importance, in such scenarios, of establishing a relationship of trust. In this sense, collaborations with non-profit organizations and/or trusted health professionals could be a key factor. Vaccination programs should also not be utilized by authorities to detect irregular immigrants. It has been suggested that governments might take advantage of the state of emergency imposed by the pandemic in order to justify control policies on the migrant and stateless population [22]. In the Mediterranean countries, it was underlined how the quarantine measures imposed by the state of emergency justified policies of migrants’ confinement otherwise not acceptable under normal conditions. Quarantine ships were classified more as floating hotspots than as medical devices [5]. Another example comes from Sicily, where it was reported that mainland hotspots made it possible to speed up expulsion policies by virtue of the emergency state dictated by the pandemic [23]. The time of quarantine, geographical segregation within isolation spaces, and selection procedures are more functional to the governance of migration than healthcare [23]. Close cooperation between the health sector and migrant protection associations [24], as well as transparent communication, would be necessary to avoid an exacerbation of distances and barriers. On a positive note, the pandemic and vaccination campaign could be an opportunity to improve integration programs, ramp up regularizations, and improve migrants’ access to public health. Portugal has provided citizenship and full access to the national health service to asylum seekers [25], while Germany has witnessed several acts of solidarity toward the most vulnerable populations [26]. Unfortunately, these remain isolated examples [7,24]. A potential benchmark for the effectiveness of the welfare and vaccination program could be the rate of vaccinated migrants, keeping faithful to the principle “no one is safe, until everyone is safe.” [14].

### 1.5. The Importance of Communication

The *Framework* [17] suggests some practical approaches to reduce vaccination barriers among the foreign population, which should be applied from the early stages of the vaccination campaign. It emphasizes the need to provide information regarding the vaccine in the mother language and in a way that is appropriate to each socio-cultural and educational context. The entity providing the information should be perceived both as reliable and socially close to the interlocutors. Ease of access to vaccination centers and, when possible, home vaccinations should be pursued. *Framework* [17] also suggests the direct involvement of members of these communities in the vaccination campaign, as well as establishing a relationship of trust and collaboration between health organizations and key figures in the target communities. Ideally, these relationships would already have been in place before the pandemic.

### 1.6. Objectives

In this study, carried out as a result of a collaboration with MEDU, a non-profit association aimed at bringing healthcare to vulnerable populations, we provide important data related to the vaccination campaign carried out in the informal settlements of Rome. This article is intended to be a primarily descriptive work in which we provide qualitative results about the field experience and quantitative data relating to vaccination coverage. The objectives of this work are to (1) evaluate vaccination coverage in the migrant and refugee population of the informal settlements in Rome, (2) assess if there is a gap with the vaccination coverage of the Italian population and hypothesize the causes, and (3) hypothesize the response that humanitarian associations can give to reduce this gap.

## 2. Methods

### 2.1. Data Sources

Medici per i Diritti Umani (MEDU; “Doctors for Human Rights”—Italy) is a non-profit humanitarian organization with the purpose of providing healthcare to vulnerable people [27]. The data shown below were collected within MEDU ‘s medical and welfare activities during the Italian vaccination campaign between 1 June 2021 and 30 September 2021. Written informed consent was given by each patient, and data relating to gender, age, nationality, legal status, intent to remain in Italy, and vaccination status were collected.

### 2.2. Study Area

**Figure 1:**
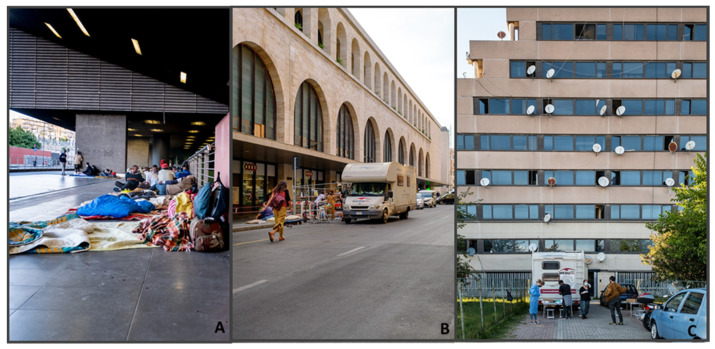
Study area: informal settlement of Rome: (**A**) Tiburtina station, homeless migrants living near the station entrance; (**B**) Termini station, MEDU’s mobile clinic parked near the station during the weekly assistance activities; (**C**) Collatina’s settlement, MEDU’s staff positioned at the foot of the building just before the weekly assistance activity. Photographs by *Odino Vignali*.

**Figure 2:**
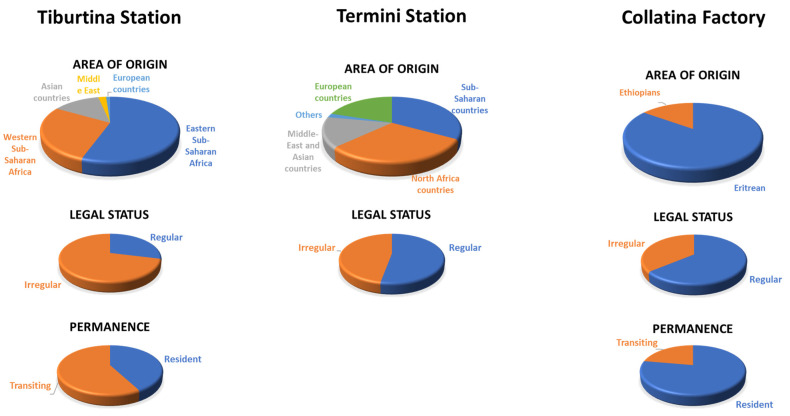
Population characteristic of the informal settlement of Rome. Area of origin, legal and permanence status vary depending on the type of settlement. Tiburtina settlement is made up mostly of transiting irregular migrants from sub-Saharan Africa. Termini station has a greater variability regarding the country of origin and the legal status of the population. In this settlement, visits were made only on the resident population. In Collatina, population comes from Ethiopia or Eritrea, and there was a higher percentage of regular and permanently resident populations than the other two settlements.

The research area covers the informal settlements of Rome, the capital of Italy, the first western state to face the COVID-19 emergency in 2020, and one of the first in the world to experiment with measures to slow the pandemic. Three informal settlements were studied—the open spaces next to the two largest train stations (Tiburtina (Tb) and Termini (Te) stations) and one of the largest occupied factories of the city (Collatina (C)) (Figure 1). Population characteristics of the informal settlement are shown in Figure 2. In total, 160 visits were carried out by MEDU’s doctor (90 in Tb, 34 in Te, and 36 in C).

#### 2.2.1. Tiburtina Station

**Tb** hosted large groups of migrants (with or without a residence permit), holders of international protection, asylum seekers, and refugees. The number of people, depending on the period of the year, ranged between 60 and 100. The settlement was evacuated at the end of July 2021, but part of the resident population continued to live there, followed by the MEDU staff in a nearby square where the data continued to be collected. During the assistance activities, 90 people were visited (89 male and 1 female), with a median age of 24.9. Overall, 42.2% (n.38) of people are permanent residents in the settlement, while 57.8% (n.52) are transiting to other European countries.

**Area of origin**: In total, 50% came from countries of Eastern sub-Saharan Africa (Eritrea, Ethiopia, Somalia, and Sudan), 25% from western sub-Saharan Africa (Burkina Faso, Ivory Coast, Chad, Gambia, Mali, Niger, Nigeria, Cameroon, Guinea, Senegal), 12% from Asian countries (Afghanistan, India, Pakistan), 10% from North Africa (Egypt, Libya, and Tunisia), 2% from the Middle East (Iran and Palestine), and 1% from European countries.

**Legal status:** Overall, 71.1% (n.64) of visited people were irregular, i.e., without any valid residence documents, while 28.9% (n.26) of visited people were regular, mostly holders of international protection, asylum seekers, or people in the process of appealing against the denial of international protection.

#### 2.2.2. Termini Station

**Te** and surrounding areas hosted between 150 and 200 homeless people. The population was characterized by extreme heterogeneity both in terms of geographical origin and legal status along with the problems encountered. During the assistance activities, 34 people were visited (32 male and 2 female), with a median age of 38.7. The totality of the people visited are permanent residents in the settlement.

**Area of origin**: Here, 33% of the population came from in sub-Saharan countries, 30% from North Africa, 15% from the Middle East and Asia, and the remainder came from other countries.

**Legal status**: Overall, 47.1% (n.15) of the people were without any regular documents, while 52.1% (n.18) of visited people were regular, among whom were people from the European community, asylum seekers, and holders of residence permits for subsidiary protection or humanitarian protection.

#### 2.2.3. Collatina Factory

The building is one of the most populous residential occupations in Rome. About 450 people currently live in the building, located on the eastern outskirts of the city. In almost all cases, they are refugees and holders of international protection of Eritrean and Ethiopian nationality, including about 30 families with minors. During the assistance activities, 36 people were visited (21 male and 15 female), with a median age of 40.17. Of those, 77.8% (n.28) are permanent residents in the settlement, while 22.2% (n.8) are transiting to other European countries.

**Area of origin**: Here, 85% were of Eritrean origin, and the remaining 15% were Ethiopian.

**Legal status:** Overall, 36.1% (n.13) of the people were without any regular documents, while 63.9% (n.23) of visited people were regular.

### 2.3. Data Collection

#### 2.3.1. Qualitative Data Collection

##### Fieldwork

The MEDU organization has been active in Rome since 2004, and one of its first projects is a mobile clinic that aims to facilitate access to healthcare for vulnerable groups such as marginalized populations, migrants, ethnic minorities, and groups with low socioeconomic status and low income. Free consultations, socio-administrative assistance, and orientation for public health access were provided in weekly sessions. This has allowed the establishment of a relationship of respect and trust with the inhabitants of Rome’s informal settlements. During the pandemic, MEDU welfare activities remained in place but with a strong focus on COVID-19 screenings, the supply of PPE and disinfectants, and training on anti-contagion measures. MEDU staff members have performed weekly visits to each informal settlement. The visits lasted about 3 h during which a part of the staff (visits team) carried out free medical visits, socio-administrative support, and orientation for access to the public health system; the other part of the staff (screening team) moved within the settlement to monitor people for suspicious COVID-19 symptoms (fever, cough, anosmia, ageusia, gastrointestinal symptoms, etc.), to provide PPE, and to educate on anti-contagion measures. Starting from March 2021, these activities began to be strongly connected to the vaccination campaign.

##### Weekly Meetings

Based on daily experiences and assessments, a weekly meeting was organized involving MEDU staff, local health workers, and other associations that provide assistance to vulnerable people. The data emerging from the meetings (reported in Section 3.1.“Our experience”) helped to optimize the approach to the vaccination campaign in informal settlements.

#### 2.3.2. Quantitative Data Collection

Data were collected during the visits using Microsoft Excel and were analyzed using Stata/IC 14.2 (StataCorp LLC, 4905 Lakeway Drive, TX, USA). People were categorized on the basis of vaccination status (vaccinated and unvaccinated), legal status (regular and irregular), and permanence in the settlement (residents or transiting). People with reserved vaccination were placed in the vaccinated group. Pearson’s chi-squared test was performed to detect differences in vaccination status between different groups. A value of *p* < 0.05 was considered significant.

## 3. Results

### 3.1. Our Experience

#### 3.1.1. Practical Approaches to Reduce Barriers

We want to highlight the importance of a clear understanding of the diverse attitudes and practices of these heterogeneous groups, and to encourage a policy based on collaboration.

Factors that can hinder the acceptance of vaccines can be as diverse as the alleged inclusion of pork derivatives, problematic for the Muslim population, or the alleged risk of autism perpetuated by misinformation [28,29]. It is, therefore, necessary to identify the beliefs among the immigrant population and residents of informal settlements that are opposed to vaccination [18]. During our activity, we encountered hostility related to religious beliefs, particularly among the elderly and religious populations. In such cases, involvement of the local religious authorities is a key step, but more in general, communication and a relationship of trust become fundamental. To this end, several “information days” on vaccination and on the measures to be taken to prevent contagion were organized in the informal settlements.

A specific finding that resulted from our experience is a greater reticence to vaccination of the sub-Saharan population (mostly Nigerians) and eastern Europeans (mostly Romanians and Ukrainians). In terms of locations, settlements close to stations (Te and Tb) were the ones where we encountered more difficulties, compared with occupied factories, with the likely reason that the former group is mostly composed of migrants in transit who lack the component of trust that can be established in time with the staff of the vaccination campaign and are generally in worse economic conditions. Additionally, difficulties in reaching the vaccination centers, especially by the elderly, can be a key factor. This highlights the need for greater care and resources for an effective vaccination campaign in these types of settlements or populations.

#### 3.1.2. Communication

**Figure 3:**
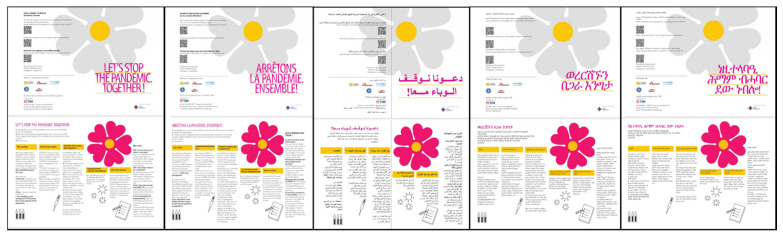
Vaccine information leaflets distributed in the informal settlements. From right to left: English, French, Arabic, Tigrinya, and Amharic. The leaflets were distributed according to the predominant language in the informal settlement. Leaflets in English and French were mainly distributed at the stations, while in the Collatina settlement, the leaflets distributed were mostly in Tigrinya and Amharic.

During the vaccination campaign, MEDU teams followed the guidelines recommended by the *Framework*. Starting from May 2021, information leaflets on the vaccine were distributed in informal settlements. Based on the most recent scientific evidence, the information leaflets were constructed and designed by MEDU in collaboration with other support associations (Médecins Sans Frontières, INTERSOS, Caritas, Sanità di frontiera, Medici del mondo) and were viewed and validated by a local health authority (Azienda Sanitaria Locale Roma 2) and translated into 10 languages by mediators (Figure 3).

The involvement of people from the settlements proved fundamental in our campaign. In particular, in Collatina, some inhabitants are also members of the MEDU staff and were involved in initiatives in which individuals with a lack of mobility were accompanied to the nearest vaccination center.

As underlined by the guidelines of the *Framework*, it is important that there is a relationship of trust between the bodies that carry out the vaccination campaign and the population in question. In the case of our experience, the MEDU staff acted in settlements, where they had been operating with healthcare services and social support for a long time.

Informal settlements such as occupied buildings or stations are difficult realities to interact with, as people are often reluctant to be helped. For healthcare workers and welfare association members, a period of time for settling and becoming accustomed to the unique situations in these areas is indispensable.

#### 3.1.3. Involvement of Local Committee

Occupied building C relies on an internal organizing committee composed of the most authoritative figures within the settlement, who, in turn, have a great influence on the population’s opinion. Since the beginning, MEDU has tried to involve the committee in decisions concerning the vaccination campaign and measures aimed at preventing the infection.

### 3.2. Vaccination Coverage in the Informal Settlements

**Scheme 1:**
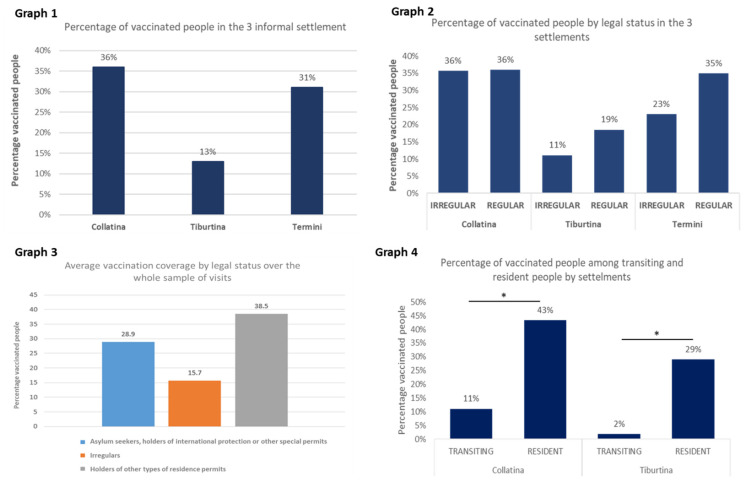
Vaccination coverage in the informal settlements: (**Graph 1**) average vaccination coverage by settlement; (**Graph 2**) vaccination coverage by legal status in the 3 settlements; (**Graph 3**) average vaccination coverage by legal status over the whole sample of visits; (**Graph 4**) vaccination coverage among transiting and resident people by settlements. (* = *p* < 0.05, CIs 95%).

The percentage of vaccinated people in the three informal settlements is very low. For comparison purposes, the vaccination coverage of the Italian population as of 30 September according to the official government website was 79.06% [30]. In Tb, we found the lowest average vaccination coverage (13.3%). In Te, the vaccination coverage was 31.4%. In C, we found the highest average vaccination coverage (35.9%) (Scheme 1, Graph 1).

In all three settlements, the irregular population had lower vaccination coverage than the regular population (11.1% vs. 18.5% in Tb; 23.1% vs. 35% in Te; 35.7% vs. 36% in C), but the differences were not significant (Scheme 1, Graph 2). Comparing the average vaccination coverage by legal status over the whole sample of visits, we found that the irregular population had the lowest vaccination coverage (15.7%). The group of asylum seekers and holders of international protection or other special permits had vaccination coverage of 28.9%, while holders of other types of residence permits had vaccination coverage of 38.5% (Scheme 1, Graph 3).

On the contrary, transiting state was significantly correlated to a lower vaccination coverage, both in Tiburtina (1.9% vs. 28.9%; *p* < 0.05, CIs 95%) and in Collatina (11.1% vs. 43.3%; *p* < 0.05, CIs 95%). Since all the population of Termini station is stable, we do not have the comparison data (Scheme 1, Graph 4).

## 4. Discussion

As the pandemic has now reached every continent, and the related deaths have exceeded 4 million, it is clear that vaccines should be considered a global public good [1]. Documents on migration highlight refugees, asylum seekers, the homeless, and foreign migrants as high-risk populations, which should, therefore, be recipients of stronger protection during the pandemic. The COVID-19 pandemic constitutes an important risk factor for populations living in informal settlements, both for the increased risk of infection and because of higher barriers in accessing health services. Furthermore, pre-existing social inequalities and vulnerabilities are exacerbated in times of emergency [9,29]. The recommendations of the *Framework* highlighted some key points, which we shared and applied when dealing with guaranteeing the rights of vulnerable people, such as the importance of establishing a relationship of trust, focusing on effective communication, involving local committees in decisions relating to the vaccination campaign, and encouraging collaborations between the national health service and welfare associations, preferably with a relationship already present before the advent of the pandemic.

With this work, we gathered important data related to the vaccination campaign carried out in the informal settlements of Rome. This study certainly has some limitations. It aims to primarily perform a qualitative and descriptive analysis and provide some data on the topic. Furthermore, according to the way in which data were collected and the possible bias in the sample population (population who visited the mobile clinic of MEDU), we cannot make inferences and comparisons to the general vaccination coverage of Italy. Additionally, in the study, the data were collected during the period of June–September and may not reflect the exact vaccination coverage at the end of September. Nonetheless, the data we collected and analyzed showed an extremely low vaccination coverage in the three settlements studied, and an important gap with the mean Italian coverage is noticeable.

In analyzing data of vaccination coverage by legal and permanence status, we found that in all the contexts studied, the transiting population has a lower vaccination coverage than that of permanent residents. These data can be explained by the impossibility of establishing a relationship of trust over time between the transiting population and the MEDU; furthermore, it is possible that a person in transiting state may be intrinsically at-risk for limited access to care. Even the irregular population showed lower vaccination coverage than the regular one, although the difference was not significant.

We also observed important differences in vaccination coverage depending on the type of settlement. We can hypothesize that inhabitants of Collatina, which has a greater percentage of regular and permanent resident people, favored greater ease in accessing care, thereby leading to the highest vaccination coverage. As indicated by our results, the difference between the transiting and resident populations was significantly different.

In Tiburtina, it is possible that the higher percentage of irregular and transiting migrants and the social hardship of living conditions led to low access to vaccination.

In Termini station, characterized by a great heterogeneity relative to the country of origin and social and legal status, we observed a higher vaccination coverage, compared with the Tiburtina station. We can hypothesize that these results can be at least partially explained by the higher percentage of permanent residents and the more central location of Termini versus that of Tiburtina may have played a role in the greater access to vaccination in the context of Termini.

## 5. Conclusions

In conclusion, through the analyses in this paper, we can provide recommendations that integrate the MEDU’s fieldwork experience with the advice of the *Framework* and can illustrate our results with data.

It must be emphasized how important it is to establish a relationship of trust with vulnerable populations and primarily with local committees if present. Furthermore, a collaboration between humanitarian associations and the local health authorities should be promoted. The data also show that particular attention must be paid to transiting and irregular populations, as they are more at risk of lacking access to vaccination. We believe that MEDU, operating in the capital since 2004, has been able to integrate the recommendations of the *Framework* within their own experience in an exemplary way. We hope that this paper will help those who work in similar contexts to have a deeper knowledge of the topic and to carry out a fair and effective vaccination campaign.

## Data Availability

We declare no competing interests.
